# Exploring the Histopathological Landscape of Urinary Bladder Diseases: A Tertiary Care Center Study

**DOI:** 10.7759/cureus.64557

**Published:** 2024-07-15

**Authors:** Aakriti Kundlia, Arpana Dharwadkar, Charusheela Gore, Vidya Viswanathan, Yaminy Ingale

**Affiliations:** 1 Pathology, Dr. D. Y. Patil Medical College, Hospital and Research Centre, Dr. D.Y. Patil Vidyapeeth, Pune, Pune, IND

**Keywords:** cystitis, non-neoplastic, neoplastic, tumours, cystoscopy, biopsy, histopathology, urothelial carcinoma, bladder carcinoma, urinary bladder

## Abstract

Introduction

Urinary bladder lesions encompass a wide spectrum, from benign inflammatory conditions to malignant neoplasms, presenting diagnostic and therapeutic challenges. Urothelial carcinoma predominates among bladder malignancies, exhibiting diverse clinical presentations and prognoses.

Objective

This study aimed to delineate the histopathological spectrum of urinary bladder lesions and correlate demographic profiles, clinical features, and cystoscopic findings with various bladder lesions.

Methods

This prospective descriptive observational study spanned 24 months at a tertiary care center, involving 65 cases of urinary bladder biopsies, including transurethral resection of bladder tumors, cystoscopic biopsies, and cystectomy specimens. The histopathological examination followed the WHO 2022 classification of urinary bladder tumors and the American Joint Committee on Cancer eighth edition staging. Clinical data, including age, gender, cystoscopic findings, and presenting symptoms, were correlated with histopathological diagnoses to explore the spectrum of bladder lesions.

Results

Neoplastic lesions predominated, constituting 92.3% of cases, with urothelial carcinoma comprising 83.33% of these cases. Among neoplastic lesions, invasive high-grade urothelial carcinoma (36.7%) and non-invasive low-grade papillary urothelial neoplasm (20.0%) were the most frequently observed subtypes. Non-neoplastic lesions accounted for 7.7%, including various forms of cystitis. Hematuria was the predominant presenting symptom (81.5%), while cystoscopic examinations revealed that most lesions were situated in the lateral bladder wall. High-grade urothelial carcinomas were mostly associated with muscularis propria invasion.

Conclusion

This study underscores the critical role of histopathological examination in diagnosing and managing urinary bladder diseases and distinguishing between non-neoplastic and neoplastic lesions. Urothelial carcinoma, prevalent among older age groups, often demonstrated muscle invasion indicative of high-grade tumors. Including the muscle layer in cystoscopic biopsies is crucial for an accurate diagnosis. Conversely, though less common, non-neoplastic conditions encompass various forms of cystitis. These findings highlight the importance of precise diagnostic tools such as cystoscopy and histopathological examination for the early detection and management of bladder neoplasms. Histopathological assessment offers essential prognostic guidance, aids in precise staging and grading, and directs tailored treatment strategies.

## Introduction

Urinary bladder lesions encompass a spectrum of infections, inflammatory conditions, metaplastic lesions, and benign and malignant tumors, contributing to notable levels of morbidity and mortality [1]. Neoplastic conditions are more commonly observed among the diverse lesions affecting the urinary bladder [2]. Urinary bladder carcinoma ranks as the second most common malignancy of the urogenital system, following prostate cancers in males [3]. According to GLOBACON 2022 data, bladder cancer ranks as the ninth most commonly diagnosed cancer, with around 614,000 new cases and 220,000 fatalities reported in 2022 [4]. Bladder tumors contribute to 6% of cancer cases in males and 2% in females [5]. In India, the incidence of bladder cancer is reported to be 20,470 cases among men and 5,403 cases among women annually. The cumulative risk is estimated at one in 250 for men and one in 1,014 for women [1,6]. Neoplasms of the bladder are more widespread in urban areas as opposed to rural regions [7]. Approximately 80% of individuals diagnosed with this condition fall within the age range of 50 to 80 years [8].

The vast majority of urinary bladder tumors are of epithelial origin, with urothelial carcinoma predominating, constituting 90% of all primary bladder tumors [3]. The majority of urothelial carcinomas in the bladder are non-invasive papillary tumors known for their slow progression but high recurrence rates following local treatment. However, approximately 20% to 30% of bladder urothelial carcinomas manifest as invasive diseases, marked by swift advancement and an unfavorable clinical prognosis [9]. Following urothelial tumors, squamous cell carcinoma (5%) and primary adenocarcinoma (2%) make up the most common types of bladder cancers. Tumors such as small cell carcinoma and sarcomas are much less commonly encountered [2,10]. The non-neoplastic lesions encompass inflammatory conditions such as different types of cystitis, tuberculosis, malakoplakia, urachal anomalies, and schistosomiasis [1,11]. Among non-neoplastic diseases, cystitis is one of the important reasons for symptomatic manifestation [11].

The biological, clinical, diagnostic, and therapeutic complexities posed by neoplastic bladder lesions present significant challenges for both urologists and pathologists, owing to the heterogeneous nature of these tumors [12]. Hematuria, which occurs in both benign and malignant cases, constitutes the primary clinical manifestation of bladder lesions [2,7]. Other symptoms include dysuria, nocturia, increased frequency, and suprapubic pain [2,13]. Patient history, clinical examination, cystoscopic evaluation, and histopathological analysis of biopsies are the fundamental components in diagnosing and treating bladder cancer [1]. Cystoscopy stands as the principal diagnostic tool for individuals suspected of bladder tumors, facilitating direct visualization of the bladder mucosa and biopsy retrieval from suspicious lesions [3,7,11,14]. However, cystoscopy alone may not always provide an accurate diagnosis, making histopathology the preferred option for the most precise and definitive diagnosis. It remains the gold standard of diagnosis.

We propose to study the comprehensive histopathological profile of bladder biopsies, ensuring a thorough evaluation.

## Materials and methods

This prospective descriptive observational study was conducted in the Department of Pathology at a tertiary care center over a 24-month period from May 2022 to May 2024. A total of 65 cases were included based on a calculated sample size using WinPepi v11.65 (Brixton Health, London, UK). Institutional ethics and scientific committee clearance was obtained from Dr. D. Y. Patil Medical College, Hospital and Research Centre Institutional Ethics Sub-committee (approval number: IESC/PGS/2022/187), and informed consent was obtained from all included patients.

Histopathological examinations were prioritized, supplemented by radiological assessments whenever possible. Biopsy specimens were collected in 10% formalin, fixed for a minimum of eight hours, processed, and embedded with the mucosal surface uppermost. The mucosal surface was kept up during embedding to ensure proper tissue orientation for sectioning and microscopic examination. Sections approximately five microns thick were cut and stained with hematoxylin and eosin. A detailed examination of multiple serial sections was performed under a light microscope, and diagnoses were rendered based on histopathological features. Special stains and immunohistochemistry were employed as necessary. Histopathological evaluations recorded details such as histologic grade, type, invasion into deeper tissues, and tumor stage, following the 2022 WHO fifth edition [15] and American Joint Committee on Cancer eighth edition cancer staging guidelines [16]. Cystoscopic studies were reviewed, and patient history, clinical findings, and cystoscopic details were correlated with histopathological results for comprehensive analysis.

Inclusion and exclusion criteria

All cystoscopic biopsies, transurethral resection of bladder tumor specimens, and cystectomy specimens received in the pathology department during the study period were included, encompassing all age groups and both genders. Autolyzed specimens, inadequate biopsies, and those lacking sufficient clinical findings were excluded from the study.

Statistical analysis

In our study, the categorical variables were presented as frequency and percentages. Data were entered into an Excel spreadsheet (Microsoft Corporation, WA, USA) and analyzed using SPSS Statistics version 20 (IBM Corp. Released 2011. IBM SPSS Statistics for Windows, Version 20.0. Armonk, NY: IBM Corp.). As this is a descriptive study, tests of statistical significance and hypothesis testing were not considered.

## Results

Among the 65 total cases in the study, the majority of patients belonged to the 61-70 years and >70 years age groups (29.2% each), followed by the 51-60 years age group (26.2%). The mean age in the study was 62.8 ± 11.7 years (Table 1).

**Table 1:** Distribution of patients according to age. The data in the table is represented as N (frequency) and % (percentage) values.

Age (in years)	Frequency (N)	Percentage (%)
<40	3	4.6
41-50	7	10.8
51-60	17	26.2
61-70	19	29.2
>70	19	29.2
Total	65	100

The highest occurrence of neoplastic lesions was observed among individuals aged 61-70 years (31.7%), followed closely by those aged over 70 years (28.3%). Non-neoplastic lesions were most commonly found in patients over 70 years old (40%), with equal representation (20%) in the <40, 41-50, and 51-60 age groups (Table 2).

**Table 2:** Age distribution of neoplastic and non-neoplastic bladder lesions. The data in the table is represented as N (frequency) and % (percentage) values.

Age (years)	Neoplastic (N, %)	Non-neoplastic (N, %)
<40	2 (3.3%)	1 (20%)
41-50	6 (10%)	1 (20%)
51-60	16 (26.7%)	1 (20%)
61-70	19 (31.7%)	0
>70	17 (28.3%)	2 (40%)
Total	60 (100%)	5 (100%)

The ratio of male to female participants in the current study was approximately 3.64:1. Most neoplastic lesions were seen in males (76.7%) compared to females (23.3%), whereas all five cases of non-neoplastic lesions were observed in males (Table 3).

**Table 3:** Gender distribution of neoplastic and non-neoplastic bladder lesions. The data in the table is represented as N (frequency) and % (percentage) values.

Gender	Type of lesion	Frequency (N)	Percentage (%)
Female	Neoplastic	14	23.3
	Non-neoplastic	0	0
Male	Neoplastic	46	76.7
	Non-neoplastic	5	100
Total		60	100

During cystoscopy, the majority of lesions were detected on the lateral bladder wall (n=33 cases) and the posterior bladder wall (n=24 cases). In our study, the majority of patients presented with hematuria (81.5%), followed by dysuria (52.3%) and burning micturition (32.2%).

**Figure 1:**
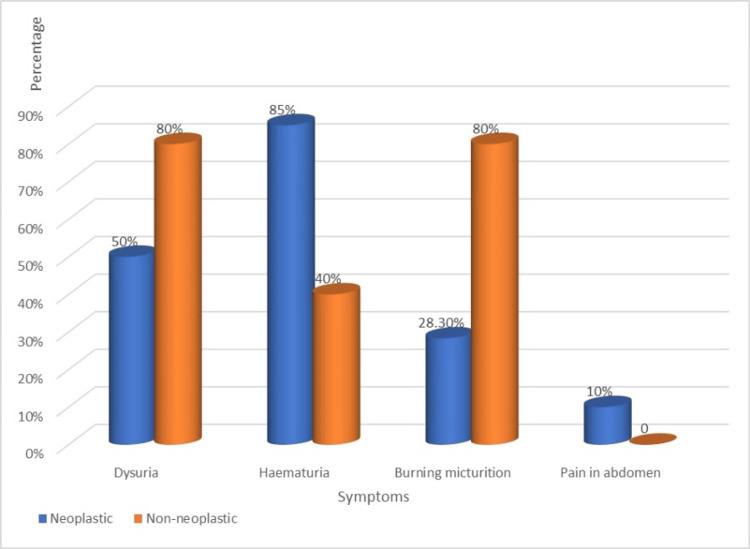
Bar graph represents the distribution of the type of lesion with symptoms.

The comparison of clinical complaints associated with neoplastic and non-neoplastic lesions yielded significant insights. Hematuria (85%), dysuria (50%), and abdominal pain (10%) were identified as prominent symptoms, with hematuria notably more prevalent in patients with neoplastic lesions (85%) compared to non-neoplastic lesions (Figure 1).

**Figure 2:**
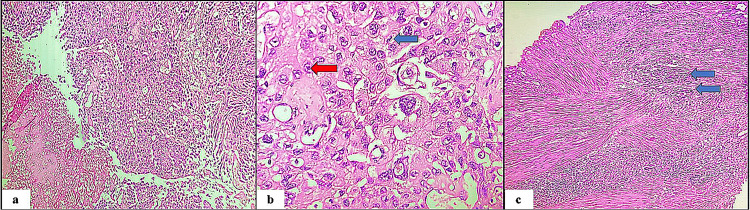
Invasive high-grade urothelial carcinoma. (a) Microscopic view demonstrating complex, solid to fused papillae with disordered architecture and crowded, overlapping cells. Areas of necrosis and subepithelial invasion were noted against a background of chronic inflammatory reactions (hematoxylin and eosin stain, x100). (b) Microscopic view displaying cells with marked nuclear pleomorphism, hyperchromatism, and nucleomegaly. Frequent prominent nucleoli (red arrow) and mitoses (blue arrow) are also noted (hematoxylin and eosin, x400). (c) Microscopic view showing tumor invading into the muscle tissue (blue arrows) (hematoxylin and eosin, x100).

**Figure 3:**
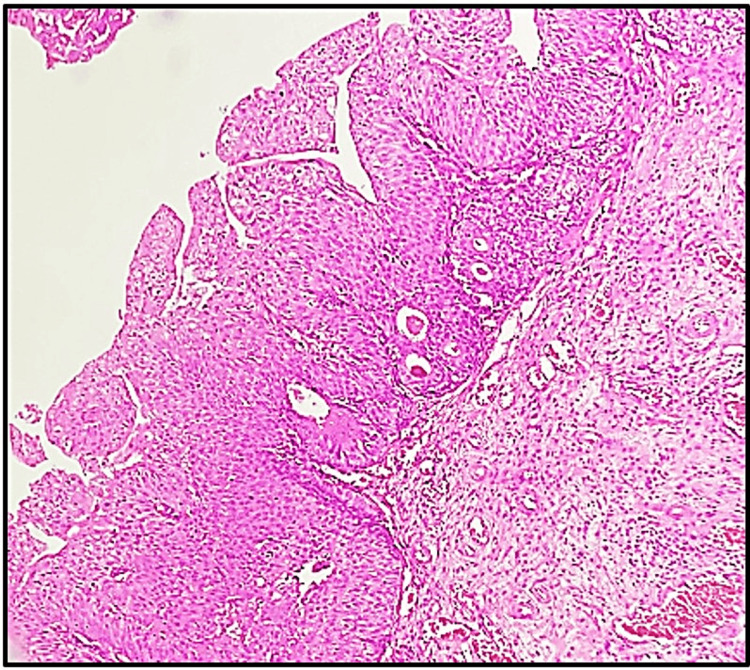
Non-invasive low-grade papillary urothelial neoplasm. Microscopic view illustrating superficial transitional epithelium forming papillae with minimal branching. The cells exhibit mild pleomorphism (hematoxylin and eosin, x100).

**Figure 4:**
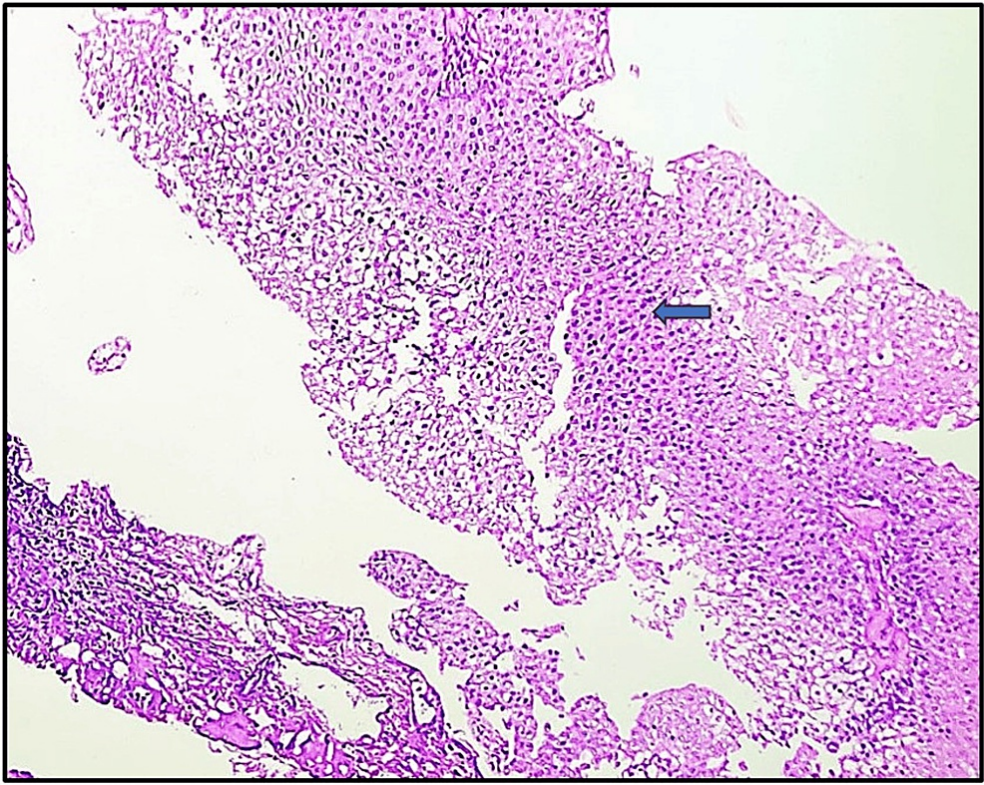
Papillary urothelial neoplasm of low malignant potential (PUNLUMP) demonstrates thickened urothelium, with the cells exhibiting a monotonous appearance and showing slight nuclear enlargement (blue arrow) (hematoxylin and eosin, x100).

In this research involving 65 cases, a significant majority (92.3%) were neoplastic lesions, with non-neoplastic lesions accounting for only 7.7% of the total cases. In the present study, among the 60 neoplastic cases analyzed, urothelial carcinoma stood out as the predominant type, comprising (n=50) 83.33% of cases. Among the 50 cases of urothelial carcinoma examined in the study, invasive high-grade urothelial neoplasm (Figure 2) emerged as the most prevalent subtype, accounting for 36.7% of cases. Following closely behind was a non-invasive low-grade papillary urothelial neoplasm (Figure 3), representing 20.0% of cases. Other noteworthy findings included non-invasive high-grade urothelial neoplasm at 8.3% and papillary urothelial neoplasm of low malignant potential (PUNLUMP) at 6.7% (Figure 4).

Table 4 shows the histopathological spectrum of various neoplastic lesions in the urinary bladder.

**Table 4:** Histopathological spectrum of various neoplastic lesions in the urinary bladder. The data in the table is represented as N (frequency) and % (percentage) values.

Neoplastic lesions	Frequency (N)	Percentage (%)
Non-invasive low-grade papillary urothelial neoplasm	12	20
Invasive low-grade papillary urothelial neoplasm	3	5
Non-invasive high-grade urothelial neoplasm	5	8.3
Invasive high-grade urothelial neoplasm	22	36.7
Invasive urothelial carcinoma with squamous differentiation	2	3.3
Poorly differentiated squamous cell carcinoma	2	3.3
Moderately differentiated squamous cell carcinoma	2	3.3
Diffuse large B-cell lymphoma (DLBCL)	1	1.7
Papilloma	2	3.3
Leiomyoma	2	3.3
Adenocarcinoma	1	1.7
Fibroepithelial polyp	2	3.3
Papillary urothelial neoplasm of low malignant potential (PUNLUMP)	4	6.7
Total	60	100

**Figure 5:**
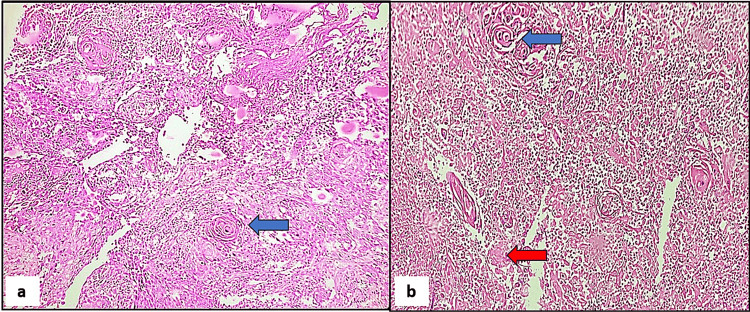
Invasive urothelial carcinoma with squamous differentiation. (a) Microscopic view showing cells arranged in irregular nests and sheets. Keratin pearls are also noted (blue arrow) (hematoxylin and eosin, x100). (b) Microscopic view showing cells arranged in irregular nests and sheets. Necrotic debris (red arrow) and keratin pearls are also noted (blue arrow) (hematoxylin and eosin, x100).

**Figure 6:**
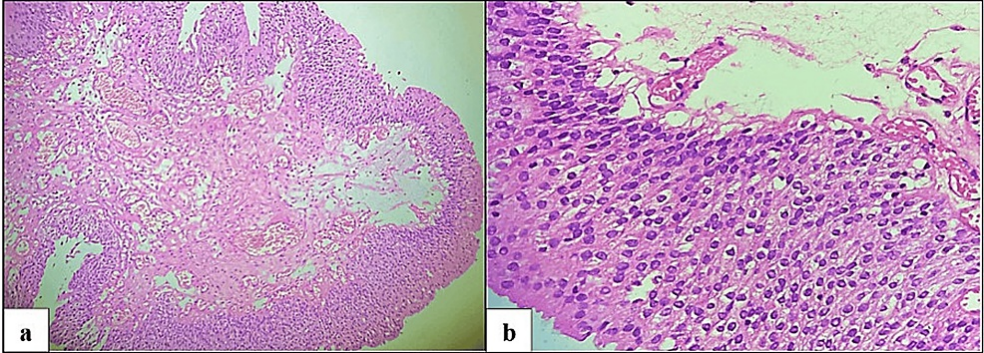
Papilloma. (a) Microscopic view demonstrating discrete, slender papillary structures with central fibrovascular cores. No fusion is noted (hematoxylin and eosin, x100). (b) Microscopic view showing the overlying urothelium with normal thickness and no cytological atypia noted (hematoxylin and eosin, x400).

Less common lesions, each constituting 3.3% of the total, included poorly differentiated squamous cell carcinoma, invasive urothelial carcinoma with squamous differentiation (Figure 5), moderately differentiated squamous cell carcinoma, papilloma (Figure 6), leiomyoma, and fibroepithelial polyp. Conversely, the least common neoplastic lesions encountered were diffuse large B-cell lymphoma (DLBCL) and adenocarcinoma, each observed in 1.7% of cases (Table 1).

**Figure 7:**
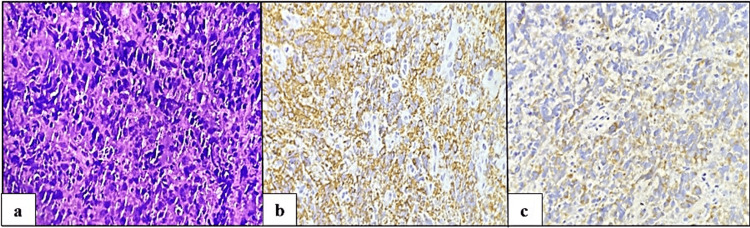
Large B-cell lymphoma (DLBCL). (a) Microscopic view demonstrating large atypical lymphoid cells (hematoxylin and eosin, x400). (b) Microscopic view showing positive CD20 expressions in atypical lymphoid cells (hematoxylin and eosin, x400). (c) Microscopic view showing positive BCL 2 expressions in atypical lymphoid cells (hematoxylin and eosin, x400).

In our research, a female patient aged 45 was diagnosed with DLBCL. Histopathological examination revealed large atypical lymphoid cells with pleomorphic nuclei, prominent nucleoli, and vesicular chromatin (Figure 7a). Immunohistochemical studies confirmed the diagnosis, showing CD20 (Figure 7b) and BCL2 (Figure 7c) positivity in the large cells.

Similarly, in our study, we observed a lower incidence of papillomas at 3.3%, with two cases identified. Additionally, we detected four cases (6.7%) of papillary urothelial neoplasms of low malignant potential (PUNLMP) (Table 1).

**Figure 8:**
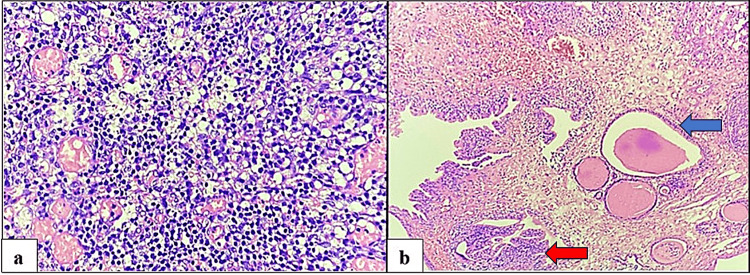
(a) Cystitis. Microscopic view showing the underlying submucosa with dense mixed inflammation composed of lymphocytes, plasma cells, and a few polymorphs. The proliferation of blood vessels is also evident (hematoxylin and eosin, x400). (b) Cystitis cystica et glandularis. Microscopic view showing numerous von Brunn nests (red arrow) and cystically dilated glands lined by cuboidal to columnar epithelium (blue arrow) (hematoxylin and eosin, x100).

In this study, non-neoplastic lesions were evenly distributed among five types: chronic cystitis, interstitial cystitis, papillary cystitis (Figure 8a), cystitis cystica et glandularis (Figure 8b), and tubercular cystitis.

**Figure 9:**
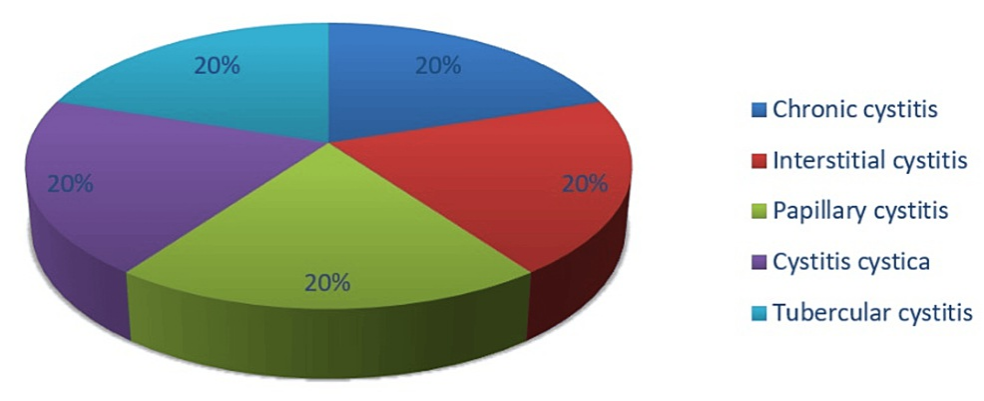
Pie graph representing the histopathological spectrum of various non-neoplastic lesions in the urinary bladder.

Each condition accounted for 20% of the total non-neoplastic lesions, with one case observed for each type (Figure 9).

Of the total 60 neoplastic lesions, 27 patients had invasive types of urothelial bladder carcinoma, with 48.1% of these patients exhibiting muscle invasion.

In our study, muscle invasion was detected in 10 cases of invasive high-grade papillary urothelial neoplasm, one case of invasive low-grade papillary urothelial neoplasm, and two cases of invasive urothelial carcinoma with squamous differentiation. In the remaining cases, invasion was limited to the lamina propria (Table 5).

**Table 5:** Presence of muscle invasion in urothelial bladder carcinoma. The data in the table is represented as N (frequency) and % (percentage) values.

Deep muscle invasion	Frequency (N)	Percentage (%)
Present	13	48.1
Absent	14	51.9
Total	27	100

## Discussion

Urinary bladder diseases can be either non-neoplastic or neoplastic [2]. The majority of non-neoplastic lesions of the urinary bladder consist of various forms of cystitis [17]. The majority of neoplastic lesions in this study were observed in the 61-70 age group (31.7%), followed by those over 70 years (28.3%) [13]. This finding is supported by studies from Lekhi et al. [13] and Hemalatha et al. [18], which also identified the 61-70 age group as the most common for bladder neoplastic lesions. Non-neoplastic lesions were most common in patients over 70 years old (40%), with equal representation (20%) in the <40, 41-50, and 51-60 age groups. Shrestha et al. [7] found the average age for non-neoplastic lesions to be 57 years, while Shanthi et al. [19] reported that non-neoplastic lesions predominantly occurred in the 31-40 age group. In fact, nearly 80% of bladder cancer cases are diagnosed in adults aged 65 or older, reflecting a disease progression that requires many years of exposure or develops later in life [20].

The male-to-female ratio in this study was approximately 3.64:1, with males comprising 78.5% of the participants. A similar male predominance was noted by Shrestha et al. [7] and Anwar et al. [21]. Most neoplastic lesions were seen in males (76.7%), with all non-neoplastic lesions observed in males. Studies by Susmitha et al. [11] and Ratnam et al. [22] also revealed a higher prevalence of neoplastic lesions in males. Conversely, Duduyemi et al. [23] indicated a higher prevalence of bladder cancers in females and found nearly equal numbers of non-neoplastic lesions between sexes.

In this study, the majority of lesions were found in the lateral bladder wall (33 cases) and posterior bladder wall (24 cases), based on cystoscopic evaluation. This finding aligns with Pandey et al.'s [1], who also identified the bladder's posterior and lateral walls as the most affected areas. Similarly, studies conducted by Jhaveri et al. [24] identified the lateral bladder wall as the most frequently involved site, accounting for 46% of cases.

In our study, the majority of patients presented with hematuria (81.5%), followed by dysuria (52.3%) and burning micturition (32.2%). This aligns with findings from Jhaveri et al. [24], who reported hematuria in 86.48% of cases, and Pandey et al. [1], who found it in 92% of cases. For neoplastic lesions, hematuria (85%), dysuria (50%), and abdominal pain (10%) were prominent, with hematuria notably more prevalent in neoplastic cases. Hemalatha et al. [18] and Shruti and Rangaswamy [25] also identified hematuria as a primary symptom in neoplastic cases. Shanthi et al. [19] observed that hematuria was the most prevalent symptom in both neoplastic and non-neoplastic cases.

Non-neoplastic lesions are often treated without biopsy and undergo histopathological examination less frequently than neoplastic lesions. They may present with milder symptoms or be asymptomatic, reducing the perceived need for a biopsy, and some respond well to conservative management. In this study, comprising 65 cases, neoplastic lesions were predominant, constituting 92.3% of cases, whereas non-neoplastic lesions were only 7.7% [1]. This observation aligns with broader trends seen in similar studies. Pandey et al. [1] reported a similarly high prevalence of neoplastic lesions at 94.7%, with non-neoplastic lesions comprising only 5.3% of cases. Additionally, Shrivastava et al. [9] and Paudel et al. [5] demonstrated a predominance of neoplastic lesions, ranging from 70.56% to 88.8%, emphasizing the significant prevalence of neoplastic bladder lesions across diverse study cohorts.

In the present study, among the 60 neoplastic cases analyzed, urothelial carcinoma was the predominant type, comprising 83.33% of cases. This finding is consistent with Shrestha and Karmacharya et al. [26], where urothelial carcinoma constituted 85.7% of cases. Similar results were reported by Jhaveri et al. [24], Shrivastava et al. [9], Ratnam et al. [22], and Paudel et al. [5], all identifying urothelial carcinoma as the most common neoplastic lesion in the urinary bladder, underscoring its prominence in bladder pathology.

Among the 50 cases of urothelial carcinoma examined, invasive high-grade urothelial neoplasm was the most prevalent subtype, accounting for 36.7% of cases, followed by non-invasive low-grade papillary urothelial neoplasm at 20.0%. Other subtypes included non-invasive high-grade urothelial neoplasm (8.3%) and papillary urothelial neoplasm of low malignant potential (6.7%). These findings align with studies by Susmitha et al. [11] and Shanthi et al. [19], which identified invasive urothelial carcinoma and invasive high-grade transitional cell carcinoma as predominant types. Similarly, research by Shrestha et al. [26] and Pandey et al. [1] highlighted the predominance of high-grade papillary urothelial carcinoma. In contrast, Jhaveri et al. [24] found invasive papillary urothelial carcinoma to be the most common neoplasm, with low-grade cases slightly more prevalent than high-grade ones. Shrivastava et al. [9] also noted a higher prevalence of non-invasive carcinomas compared to invasive ones.

Of the 60 neoplastic lesions in our study, 27 patients had invasive urothelial bladder carcinoma. Among these, 48.1% exhibited muscle invasion, with most cases being high-grade. Similarly, in a study by Lekhi et al. [13], muscle invasion was found in 77.4% of high-grade urothelial carcinoma cases, while no invasion was observed in low-grade cases. In a retrospective study by Shrestha et al. [26], transitional cell carcinoma comprised 87.5% of urothelial origin neoplastic lesions, with 45.2% showing muscle invasion, predominantly in high-grade lesions. Conversely, Jhaveri et al. [24] reported that 80% of invasive high-grade urothelial cell carcinomas exhibited muscle invasion. The presence of muscle invasion is strongly associated with high-grade tumors, highlighting the critical role of accurate histopathological assessment for prognosis and treatment planning [24].

Less common lesions, each constituting 3.3% of the total in this study, included poorly differentiated squamous cell carcinoma, invasive urothelial carcinoma with squamous differentiation, moderately differentiated squamous cell carcinoma, papilloma, leiomyoma, and fibroepithelial polyp. The least common neoplastic lesions encountered were DLBCL and adenocarcinoma, each observed in 1.7% of cases. In a study by Kumar and Yelikar [12], transitional cell carcinoma was the most prevalent tumor, constituting 93.4% of cases, followed by squamous cell carcinoma and adenocarcinoma, accounting for 6% collectively. Similarly, Duduyemi et al. [23] identified urothelial carcinoma as the predominant bladder malignancy, with squamous cell carcinoma as the second most common type.

According to research by Pudaisaini et al. [27], glandular neoplasms like adenocarcinoma and signet ring cell carcinoma accounted for 6.3% of malignant bladder lesions, while urothelial tumors comprised 93.7%. They noted a single instance of signet ring cell adenocarcinoma in an 80-year-old man, emphasizing its rarity and poor prognosis. Adenocarcinoma, an uncommon variant of urinary bladder carcinoma, was also highlighted in studies by Laishram et al. [28].

In our study, we noted a single case of DLBCL. Primary lymphoma of the urinary bladder constitutes only 0.2% of non-Hodgkin lymphomas originating outside lymph nodes, whereas secondary involvement of the bladder by systemic lymphoma is more common. Despite its rarity, DLBCL represents the predominant subtype of primary bladder lymphoma. The majority of documented cases in the bladder are classified as DLBCL unless otherwise specified [29].

In a retrospective analysis conducted by Shrestha et al. [26], benign lesions were found to be the most common, with papillomas comprising 7.1% of cases, followed by hemangiomas and PUNLMP, each representing 2.4% of the total cases. Laishram et al. [28] reported a similar distribution, with papillomas accounting for 7.7% and PUNLMP representing 3.9% of cases. Ahmad et al.'s [8] study on the histopathological spectrum of urothelial lesions documented two cases (6.66%) of papillomas. Conversely, Vaidya et al. [30] reported a lower incidence of papillomas at 0.3% and a higher prevalence of PUNLMP at 13.58%.

Similarly, in our study, we observed a lower incidence of papillomas at 3.3%, with two cases identified. Additionally, we detected four cases (6.7%) of PUNLMP.

Among the non-neoplastic lesions in our study, chronic cystitis, interstitial cystitis, papillary cystitis, cystitis cystica et glandularis, and tubercular cystitis each accounted for 20% of the total, with one case per type. In parallel studies, chronic nonspecific cystitis emerged as the most common non-neoplastic lesion in research conducted by Sushmita et al. [11] and Mainali et al. [31]. Shanti et al. [19] reported that granulomatous cystitis was the most common among non-neoplastic lesions. Shrestha et al. [26] revealed that ulcerative polypoid cystitis was the most frequent non-neoplastic lesion in their retrospective study. In Paudel et al.'s study [5], cystitis cystica was identified as the most common non-neoplastic lesion, accounting for 8% of cases, followed by urothelial hyperplasia at 4%. Pandey et al. [1] also reported a case of cystitis cystica et glandularis.

Limitations

The limitations of our study include its single-center design, which may not reflect the broader population. The retrospective nature introduces potential selection bias and limits causality determination. Inadequate biopsy sampling, particularly the exclusion of muscularis propria, could lead to an underestimation of tumor invasiveness and staging inaccuracies. The sample size may not be large enough to provide comprehensive results. Non-neoplastic lesions are less frequently sent for biopsy and histopathological examination, often due to milder or asymptomatic presentations and effective conservative management. This results in a potential underrepresentation of non-neoplastic conditions in the study.

## Conclusions

Our study emphasizes the critical role of histopathological examination in diagnosing and managing urinary bladder diseases, distinguishing between non-neoplastic and neoplastic lesions. Urothelial carcinoma was prevalent among older age groups, often demonstrating muscle invasion, indicative of high-grade tumors. Including the muscle layer in cystoscopic biopsies is crucial for an accurate diagnosis. Conversely, non-neoplastic conditions, though less common, encompassed various forms of cystitis. These findings underscore the importance of precise diagnostic tools such as cystoscopy and histopathological examination for the early detection and management of bladder neoplasms. Histopathological assessment offers essential prognostic guidance, aids in precise staging and grading, and directs tailored treatment strategies.
